# Transformation-invariant visual representations in self-organizing spiking neural networks

**DOI:** 10.3389/fncom.2012.00046

**Published:** 2012-07-25

**Authors:** Benjamin D. Evans, Simon M. Stringer

**Affiliations:** Department of Experimental Psychology, Centre for Theoretical Neuroscience and Artificial Intelligence, University of OxfordOxford, UK

**Keywords:** transformation-invariant visual object recognition, integrate and fire, spiking neural net, continuous transformation learning, trace learning, inferior temporal cortex

## Abstract

The ventral visual pathway achieves object and face recognition by building transformation-invariant representations from elementary visual features. In previous computer simulation studies with rate-coded neural networks, the development of transformation-invariant representations has been demonstrated using either of two biologically plausible learning mechanisms, Trace learning and Continuous Transformation (CT) learning. However, it has not previously been investigated how transformation-invariant representations may be learned in a more biologically accurate spiking neural network. A key issue is how the synaptic connection strengths in such a spiking network might self-organize through Spike-Time Dependent Plasticity (STDP) where the change in synaptic strength is dependent on the relative times of the spikes emitted by the presynaptic and postsynaptic neurons rather than simply correlated activity driving changes in synaptic efficacy. Here we present simulations with conductance-based integrate-and-fire (IF) neurons using a STDP learning rule to address these gaps in our understanding. It is demonstrated that with the appropriate selection of model parameters and training regime, the spiking network model can utilize either Trace-like or CT-like learning mechanisms to achieve transform-invariant representations.

## 1. Introduction

The increasingly complex cell response properties of the primate ventral visual stream strongly suggest the functional organization of this pathway is that of a feature hierarchy. Cells in the early stages (V1) are found to be sensitive to oriented bars and edges appearing in particular locations on the retina (Hubel and Wiesel, 1968). Information analysis of natural scenes reveals these features to be the most statistically independent components of such images (Bell and Sejnowski, 1997; van Hateren and van der Schaaf, 1998) and hence the most natural “building-blocks” for such a system. Through successive layers, there follows a convergence of receptive fields allowing neurons at the end of the pathway in anterior Inferotemporal cortex (aIT) to view the entire retina and respond to increasingly complex stimuli (Tanaka, 1996). Here, and more recently in the medial temporal lobe (Quiroga et al., 2005), neurons have been found which respond with translation (Op de Beeck and Vogels, 2000), size (Ito et al., 1995) and view invariance (Booth and Rolls, 1998) to objects (Tanaka et al., 1991) and faces (Desimone, 1991).

Several groups have attempted to understand how elementary features may be combined into more complex view-invariant representations of whole objects with hierarchical feed-forward neural network models such as the *Neocognitron* (Fukushima, 1988), the *SEEMORE* system (Mel, 1997), the *HMAX* model (Riesenhuber and Poggio, 1999) and *VisNet* (Wallis and Rolls, 1997). These models are all composed of “rate-coded” neurons (McCulloch and Pitts, 1943) which consist of applying a non-linear function (e.g., threshold or sigmoid) to a weighted sum of inputs (Boolean, or real values) which they receive at each computational step^1^.

Within this paradigm, two main biologically plausible learning mechanisms have been discovered which explain how different views of the same object may be bound together and recognized as the same entity. The first of these—*Trace learning* (Földiák, 1991)—relies upon temporal continuity, while the second—*Continuous Transformation (CT) learning* (Stringer et al., 2006)—relies upon spatial continuity to associate together successive transforms and build view-invariant representations in later layers. While the properties of these mechanisms have been explored extensively in rate-coded models, it remains an open question as to how they might map onto a more biologically realistic spiking-neuron paradigm.

Spiking Neural Networks (SNN) can solve problems at least as complex as those that rate-coded models can solve (Šíma and Orponen, 2003), which in turn have greater computational power than Turing machines, and as such have been applied to a wide variety of problems, including modeling object recognition (Michler et al., 2009). By more faithfully modeling the electrical properties of neurons, spiking neural network model parameters may be more meaningfully mapped onto the biophysical properties of their real counterparts. This motivates the use of the conductance-based “leaky” integrate-and-fire (LIF) model (described in section 2) over models which are computationally cheaper or have a less apparent correspondence to measurable biological parameters such as the Spike Response Model (Gerstner and Kistler, 2006) or Izhikevich's null-cline derived model (2003).

Since time is explicitly and accurately modeled in SNNs, they allow quantitative investigation of the time-course of processing on such tasks (Thorpe et al., 2000) providing further arguments against rate-coding on the basis that Poisson rate-codes are too inefficient to account for the rapidity of information processing in the human visual system^2^ (Thorpe et al., 1996; Rullen and Thorpe, 2001). Furthermore, SNNs allow the investigation of qualitative effects such as the selective representation of one stimulus over another by the synchronization of its population of feature-neurons as found in neurophysiological studies (Kreiter and Singer, 1996; Fries et al., 2002). Similarly, the phenomenon of Spike-Time Dependent Plasticity (STDP) and its effect upon learning transformation-invariant representations may only be investigated by modeling individual spikes which is of great importance to the present research.

Hebb originally conjectured that synapses effective at evoking a response should grow stronger (Hebb, 1949), capturing a causal relationship between the two neurons. This was eventually simplified (partly for the purposes of rate-coded models) to become interpreted as any long-lasting synapse-specific form of modification dependent upon correlations between presynaptic and postsynaptic firing. This is usually expressed in the form δ *w*_*ij*_ = *ky*_*i*_*x*_*j*_, where δ *w*_*ij*_ is the change in synaptic strength, *k* is a learning rate constant, and *x*_j_ and *y*_*i*_ are the firing rates of the presynaptic and postsynaptic neurons (see e.g., Rolls and Treves, 1998).

Progress in neurophysiology has shown, however, that the all-or-nothing nature of an action potential means that the information may be conveyed by the number *and* the timing of action potentials (Ferster and Spruston, 1995; Maass and Bishop, 1999), typically neglecting their size and shape in modeling. In other words neurons communicate by a *pulse* code (a time series of discrete binary events) rather than simply a *rate* code (a moving average level of activity) which has been convincingly demonstrated in the sensory systems of several organisms, such as echolocating bats (Kuwabara and Suga, 1993) and the visual systems of flies (Bialek et al., 1991).

It is also now well-established that *synaptic plasticity* is sensitive to the relative timing of the presynaptic and postsynaptic spikes (Markram et al., 1997; Dan and Poo, 2006), typically becoming approximately exponentially less sensitive as the time difference increases (Bi and Poo, 1998). This has been found to take several forms in different brain regions (Abbott and Nelson, 2000) but here we focus on the form observed in retinotectal connections and neocortical and hippocampal pyramidal cells where *pre* → *post* spike pairs lead to synaptic potentiation (with greater effect over shorter intervals) and the opposite ordering of spikes leads to synaptic depression.

The challenge now is to investigate how the timing of spikes affects the self-organization of the system applied to the problem of developing transformation-invariant representations and understanding how the CT and Trace learning mechanisms, which have been developed in the context of rate-coded models, might fit into a model of STDP.

## 2. Methods

### 2.1. Network architecture

While the ventral visual stream is typically modeled as four or more layers of neurons with excitatory modifiable feed-forward synapses and a mechanism of lateral inhibition, here we seek to understand the mechanisms operating at each layer which ultimately may lead to transformation-invariant representations, hence a simpler architecture is used.

**Figure 1:**
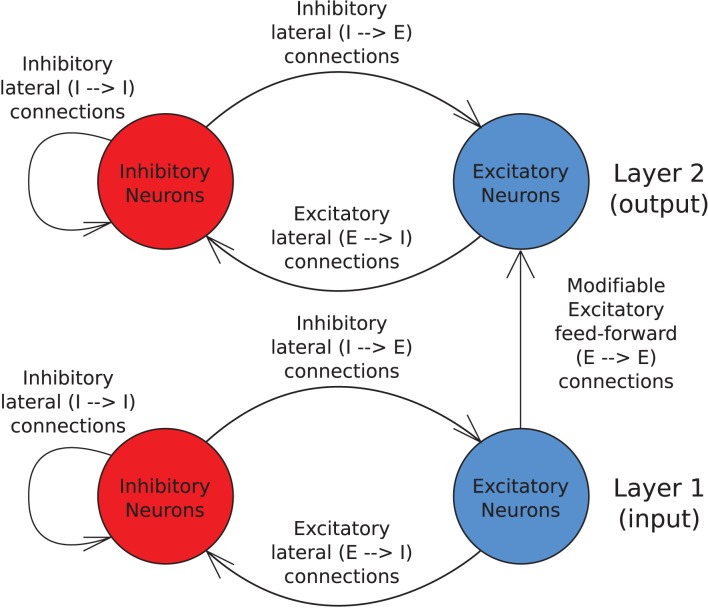
**Schematic of the two layer network architecture used.** Excitatory neurons within each layer are fully reciprocally connected to the pool of inhibitory interneurons within the same layer by fixed synaptic efficacies. Inhibitory neurons are also fully connected to other inhibitory neurons within the same layer by unmodifiable synapses. The excitatory neurons in the input layer are fully connected to the excitatory neurons in the output layer via plastic feed-forward synapses which are modified through training by an STDP learning rule.

The model consists of two layers of excitatory pyramidal neurons with one layer of modifiable feed-forward synapses between them (as shown in Figure 1). Within each layer there are also inhibitory interneurons with non-plastic lateral synaptic connections to and from the excitatory neurons to produce a degree of competition between the excitatory neurons.

For all presented simulations we have used 400 excitatory neurons and 100 inhibitory neurons in each layer, with full connectivity. Each neuron is based upon the standard conductance-based leaky integrate and fire (LIF) model (see for example Rolls and Treves, 1998) while the equations for STDP at the Excitatory-Excitatory (*E* → *E*) synapses are adapted from Perrinet et al. (2001).

### 2.2. Differential equations

#### 2.2.1. Cell equations

Depolarization of the neuron's membrane potential is described by Equation 1 and the cell (and synapse) constants were chosen to be as biologically accurate as possible based upon the available neurophysiological literature (see Table 1 for a full list).

**Table 1:** **Parameters used in the simulations**.

**Parameter**	**Symbol**	**Value**	**Reference**
Cue current	*I*^*ext*^	1.0 nA	^*^
Cue period {training, testing}	*t*_*cue*_	{100, 250}ms	
Time step	Δ*t*	0.02ms	
**NETWORK PARAMETERS**			
No. of layers	*N*_*L*_	2	
No. of excitatory cells per layer	*N*_*E*_	400	
No. of inhibitory cells per layer	*N*_*I*_	100	
No. of afferent excit. connections per excit. neuron	*S*_*EE*_	400	
No. of afferent excit. connections per inhib. neuron	*S*_*EI*_	400	
No. of afferent inhib. connections per excit. neuron	*S*_*IE*_	100	
No. of afferent inhib. connections per inhib. neuron	*S*_*II*_	100	
**CELLULAR PARAMETERS**			
Excitatory cell somatic capacitance	*C*^*E*^_*m*_	500 pF	§
Inhibitory cell somatic capacitance	*C*^*I*^_*m*_	214 pF	§
Excitatory cell somatic leakage conductance	*g*^*E*^_0_	25 nS	§
Inhibitory cell somatic leakage conductance	*g*^*I*^_0_	18 nS	§
Excitatory cell membrane time constant	τ^*E*^_*m*_	20ms	§
Inhibitory cell membrane time constant	τ^*I*^_*m*_	12ms	§
Excitatory cell resting potential	*V*^*E*^_0_	−74mV	§
Inhibitory cell resting potential	*V*^*I*^_0_	−82mV	§
Excitatory firing threshold potential	Θ^*E*^	−53mV	§
Inhibitory firing threshold potential	Θ^*I*^	−53mV	§
Excitatory after-spike hyperpolarization potential	*V*^*E*^_*H*_	−57mV	§
Inhibitory after-spike hyperpolarization potential	*V*^*I*^_*H*_	−58mV	§
Excitatory reversal potential	${\hat{V}}^{E}$	0mV	§
Inhibitory reversal potential	${\hat{V}}^{\text{I}}$	−70mV	§
Absolute refractory period	τ_*R*_	2ms	§
**SYNAPTIC PARAMETERS**			
Synaptic neurotransmitter concentration	α_*C*_	0.5	†
Proportion of unblocked NMDA receptors	α_*D*_	0.5	†
Presynaptic STDP time constant	τ_*C*_	[3, 75] ms	†
Postsynaptic STDP time constant	τ_*D*_	[5, 125] ms	†
Synaptic learning rate	ρ	0.1	†
Plastic (*E* → *E*) synaptic conductance range, CT	λ·Δ*g*^*EE*^	[0, 4] nS	^*^
Plastic (*E* → *E*) synaptic conductance range, Trace	λ·Δ*g*^*EE*^	[0, 1.25] nS	^*^
Change in synaptic conductance (*I*→ *E*)	λ·Δ*g*^*IE*^	[0.5, 2.5] nS	^*^
Change in synaptic conductance (*E*→ *I*)	λ·Δ*g*^*EI*^	5.0 nS	^*^
Change in synaptic conductance (*I*→ *I*)	λ·Δ*g*^*II*^	5.0 nS	^*^
Excitatory-Excitatory synaptic time constant	τ_*EE*_	{2, 150}ms	^*^
Inhibitory-Excitatory synaptic time constant	τ_*IE*_	5ms	§
Excitatory-Inhibitory synaptic time constant	τ_*EI*_	2ms	§
Inhibitory-Inhibitory synaptic time constant	τ_*II*_	5ms	§

Most integrate and fire parameters were taken from Troyer et al. (1998) (derived originally from McCormick et al. 1985) as indicated by §. Plasticity parameters (denoted by †) are taken from Perrinet et al. (2001). Parameters marked with ^*^ were tuned for the reported simulations.

The cell membrane potential for a given neuron (indexed by *i*) is driven up by presynaptic excitatory conductances (or direct current injection) and towards the inhibitory reversal potential (typically down) by presynaptic inhibitory conductances, decaying back to its resting state over a time course determined by the properties of its membrane.

(1) $$\tau_{m}^{\gamma }\frac{dV_{i}(t)}{dt}=V_{0}^{\gamma }-V_{i}(t)+R^{\gamma }I_{i}(t)+R^{\gamma }I_{i}^{ext}(t)+\;\sigma \cdot \xi (t)\cdot \sqrt{\tau_{m}^{\gamma }}$$

Here τ_*m*_ represents the membrane time constant, defined as τ_*m*_ = *C*_m_/*g*_0_, where *C*_*m*_ is the membrane capacitance, *g*_0_ is the membrane leakage conductance and *R* is the membrane resistance, (*R*=1/*g*_0_). *V*_0_ denotes the resting potential of the cell (indexed by γ along with these other class-specific parameters), *I*_*i*_(*t*) represents the total synaptic current (described in Equation 2) and *I*^*ext*^_*i*_(t) models the injected current.

In addition, Gaussian white noise was added to the cell membrane potential with zero mean and standard deviation σ = 0.015 · (Θ − *V*_*H*_) as used by Masquelier et al. (2009). Here, ξ(*t*) is a Wiener (Gaussian) variable (where ξ(*t*) represents $\frac{dW}{dt}$) satisfying the definition of the Wiener process such that 〈ξ〉 = 0 and 〈ξ(*t*)ξ(*s*)〉 = δ(*t*−*s*), where δ(·) is the Dirac delta function and σ tunes the amplitude of the noise (the standard deviation of the noise in units of Volts) since ξ has unit variance. The noise term, ξ, is importantly scaled by (the square root) of the time constant, τ_*m*_, which means that the amplitude of the noise is scaled up or down as the system speeds up (short τ_*m*_) or slows down (long τ_*m*_), respectively. The dimension of the ξ term is *time*^−1^ and so ξ is scaled by $\sqrt{\tau_{m}}$ to make the equation dimensionally consistent.

The total synaptic conductance is the sum of conductances of all presynaptic neurons of each type (excitatory and inhibitory) with inhibitory conductances being negative.

(2) $$I_{i}(t)=\sum_{\gamma }\sum_{j}g_{ij}(t)\left({\hat{V}}^{\gamma }-V_{i}(t)\right)$$

Here $\hat{V}$ represents the reversal potential of a particular class of synapse (denoted again by γ) which consists of Excitatory and Inhibitory neurons {*E*, *I*} and *j* indexes the presynaptic neurons of each class.

#### 2.2.2. Synaptic conductance equations

The synaptic conductance of a particular synapse, *g*(*t*), (indexed by *ij*) is governed by a decay term τ_*g*_ and a Dirac delta function for when spikes occur, which correspond to the first and second terms of Equation 3. The Dirac delta function is defined as follows:

$$\delta (x)=\{\begin{array}{cc} \infty & \text{if }x=0\\ 0 & \text{otherwise} \end{array}\text{ where, }\int_{-\infty }^{+\infty }\delta (x)dx=1.$$

The conduction delay for a particular synapse is denoted by Δ*t*_*ij*_ and each spike is indexed by *l* as a separate train for each presynaptic neuron. A biological scaling constant, λ (set in all simulations to be 5 ns) has been introduced to scale the synaptic efficacy Δ*g*_*ij*_ which lies between unity and zero.

(3) $$\frac{dg_{ij}(t)}{dt}=-\frac{g_{ij}(t)}{\tau_{g}}+\;\lambda \Delta g_{ij}(t)\sum_{l}\;\delta \left(t-\Delta t_{ij}-t_{j}^{l}\right)$$

#### 2.2.3. Synaptic learning equations

The following differential equations describe the STDP occuring at each modifiable *Excitatory*−*Excitatory*(*E*→ *E*) synapse. Here *i* labels the postsynaptic neuron. The recent presynaptic activity, *C*_*ij*_(*t*), is modeled by Equation 4 which may be interpreted as the concentration of neurotransmitter (glutamate) released into the synaptic cleft (Perrinet et al., 2001) and is bounded by [0, 1] for 0 ≤ α_C_ < 1.

(4) $$\frac{dC_{ij}(t)}{dt}=-\frac{C_{ij}(t)}{\tau_{C}}+\;\alpha_{C}\left(1-C_{ij}(t)\right)\sum_{l}\delta \left(t-\Delta t_{ij}-t_{j}^{l}\right)$$

The presynaptic spikes drive *C*_*ij*_(*t*) up at a synapse according to the model parameter α_*C*_, which then the current value of *C*_*ij*_(*t*), which then decays back to 0 over a time course governed by τ_*C*_.

The recent postsynaptic activity, *D*_*i*_(*t*), is modeled by Equation 5 which may be interpreted as the proportion of unblocked NMDA receptors as a result of recent depolarization through back-propagated action potentials (Perrinet et al., 2001).

(5) $$\frac{dD_{i}(t)}{dt}=-\frac{D_{i}(t)}{\tau_{D}}+\;\alpha_{D}\left(1-D_{i}(t)\right)\sum_{k}\delta \left(t-t_{i}^{k}\right)$$

Unlike with the conduction of action potentials to postsynaptic neurons, there is no conduction delay associated with *D*_*i*_ since the cell body is assumed to be arbitrarily close to the receiving synapses, and it is the same for a given (postsynaptic) neuron rather than each of its synapses since the effects of a postsynaptic spike are assumed to have an equal impact on all receiving synapses.

The strength of the synaptic weight, Δ*g*_*ij*_(*t*), is then modified according to Equation 6, which is governed by the time course variable τ_Δ*g*_.

(6) $$\tau_{\Delta g}\frac{d\Delta g_{ij}(t)}{dt}\;=\left(1-\Delta g_{ij}(t)\right)C_{ij}(t)\sum_{k}\delta \left(t-t_{i}^{k}\right)\;-\Delta g_{ij}(t)D_{i}(t)\sum_{l}\delta \left(t-\Delta t_{ij}-t_{j}^{l}\right)$$

Note that the postsynaptic spike train (indexed by *k*) is now associated with the presynaptic state variable (*C*) and vice versa. If *C* is high (due to recent presynaptic spikes) at the time of a postsynaptic spike, then the synaptic weight is increased (LTP) whereas if *D* is high (from recent postsynaptic spikes) at the time of a presynaptic spike then the weight is decreased (LTD).

The weight updates are also multiplicative, meaning that the amount of potentiation decreases as the synapse strengthens, as has been found experimentally (Bi and Poo, 1998). Theoretically, this weight-dependent potentiation yields a normal distribution of synaptic efficacies rather than pushing each weight to one extreme or the other (van Rossum et al., 2000) as would be the case with an additive form of STDP.

### 2.3. Numerical scheme

The differential equations described above are converted to finite difference equations and simulated using the Forward-Euler numerical scheme with a time step Δ*t* = 0.02 ms. In the finite difference equations, the Dirac delta function has been replaced by the discrete approximation, *S*(*x*) as defined in Amit and Brunel (1997). Finally, in the original description, the change in synaptic weight (Equation 6) was instantaneous and so Δ*t*/τ_Δ*g*_ is defined to be a learning rate constant, ρ, in the corresponding finite difference equation.

### 2.4. Training and stimuli

**Figure 2:**
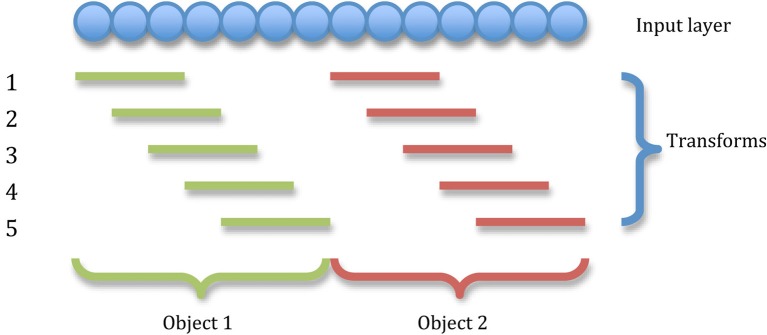
**The transforms of two stimuli.** The input layer is divided into as many equal portions as there are stimuli and all transforms of a particular stimulus are confined to that stimulus's portion of the input neurons. In this illustration, there are five transforms per object shifting by one neuron.

Stimuli are represented by injecting a small amount of current directly into the cell bodies of a particular set of excitatory input neurons continuously throughout the cue period. This pattern of stimulated neurons is gradually shifted across the input layer representing successive transforms of the stimulus (see Figure 2).

The size of a stimulus and the amount of neurons each of its transforms is shifted by allows us to precisely control the degree of overlap between transforms of each stimulus. Spatial continuity is crucial to the functioning of the CT mechanism, whereas the trace mechanism requires temporal continuity to associate successive transforms together, (which can be controlled independently through model time constants). In this way, we may eliminate the operation of one mechanism to study the other in isolation and hence disentangle their contributions to the network's capacity for invariance learning.

During training, the set of stimuli are presented in a random order with all transforms for a given stimulus being presented in succession before presenting the next stimulus's transforms. Presentation of all stimuli in this manner constitutes one training epoch, and the total training period comprised of five such epochs.

After completion of training, learning is switched off (prohibiting further synaptic modification) and the network is presented with all transforms of all stimuli in order (resetting the neurons to their resting state between transforms) and the resultant firing in both input and output layers is saved for analysis.

### 2.5. Performance measures

Two information-theoretic^3^ measures are used to assess the network's performance which reflect the extent to which cells respond invariantly to a particular stimulus over several transforms but differently to other stimuli [for more details see Rolls and Milward (2000); Elliffe et al. (2002)]. The work presented has used spiking neural networks because we believe that their richer dynamics they model are critical for learning to solve the problem of object recognition (transformation-invariant cell responses). However, analysis of macaque visual cortical neuron responses has found that after learning, the majority of the information about stimulus identity is contained within the *firing rates* rather than the detailed timing of spikes (Tovee et al., 1994). As such, we adopt a dual approach whereby the network self-organizes through spiking dynamics but the information content with respect to stimulus identity is assessed through the output cell's firing rates.

During testing each transform of each stimulus was presented to the input layer of the network. Each neuron was reset (allowed to settle) after presentation of each transform such that the activity due to one transform did not affect the responses to later transforms. After testing, the spikes of each output neuron were placed into a different bin for each transform of each stimulus and the corresponding firing rate for each cell was calculated. Based upon these firing rates, the stimulus-specific single-cell information *I*(*s, R*) was calculated according to Equation 7, which gives the amount of information in a set of responses *R* of a single cell about a specific stimulus *s*. The set of responses, *R* consisted of the firing rate of a cell to every stimulus presented in every location.

(7) $$I(s,R)=\sum_{r\in R}P(r|s)\log_{2}\frac{P(r|s)}{P(r)}$$

Good performance for a cell would entail stimulus specificity (with generality across most or all transforms of that stimulus), meaning a large response to one or a few stimuli regardless of their position (transform) and small responses to other stimuli. We therefore compute the maximum amount of information a neuron conveys about *any* of the stimuli rather than the average amount it conveys about the whole set *S* of stimuli (which would be the mutual information).

If all the output cells learnt to respond to the same stimulus then there would be no discriminability and the information about the set of stimuli *S* would be poor. To test this, the multiple cell information measure is used which calculates the information about the set of stimuli from a population of up to 10 output neurons. This population consisted of the subset of up to five cells which had, according to the single cell measure, the most information about each of the two stimuli. Ideally, we would calculate the mutual information (the average amount of information about which stimulus was shown from the responses of all cells after a single presentation of a stimulus, averaged across all stimuli), however, the high dimensionality of the neural response space and the limited sampling of these distributions is prohibitive.

Instead, a decoding procedure is used to estimate the stimulus *s*′ that gave rise to the particular firing rate response vector on each trial. From this a probability table is then constructed of the real stimuli *s* and the decoded stimuli *s*′, from which the mutual information is calculated (Equation 8).

(8) $$I(s,s^{\prime })=\sum_{s,s^{\prime }}P(s,s^{\prime })\log_{2}\frac{P(s,s^{\prime })}{P(s)P(s^{\prime })}$$

A Bayesian decoding procedure is used for this purpose, whereby the firing rates of each cell in the ensemble vector to each transform of each stimulus in turn is fitted to a Gaussian distribution parameterized by these means and standard deviations of each cell's responses to all other transforms of each stimulus separately to yield an estimate of *P*(*r*_*c*_|*s*′). Taking the product of these probabilities over all cells in the response vector with *P*(*s*′) and then normalizing the resultant joint probability distribution gives an estimate of *P*(*s*′|*r*), (Földiák, 1993). These probability distributions are factored into a confusion matrix of *P*(*s*,*s*′) over many iterations to smooth the effects of randomly sampling the output cells. From this decoding and cross-validation procedure, the probability tables are constructed for calculating the multiple cell information measure, further details of which may be found in Rolls et al. (1997). This measure should increase up to the theoretical maximum *log*_2_*N*_*S*_ bits, (where *N*_*S*_ is the number of stimuli), as a larger population of cells is used, only if those cells have become tuned to different stimuli.

## 3. Simulations

In the simulations described below we investigated invariance learning in a spiking neural network with STDP utilizing two different learning mechanisms. For details of the methods and parameters used for the following simulations, please refer to section 2 and Table 1, respectively.

### 3.1. Continuous transformation learning

Continuous Transformation (CT) learning relies upon the spatial continuity of continuously transforming stimuli and a purely associative (Hebbian) learning rule with lateral competition to associate together successive transforms of a stimulus (Stringer et al., 2006). Presentation of an initial transform will excite one or more postsynaptic neurons and through the Hebbian learning rule, will strengthen the synapses between those cells. If there is enough overlap (similarity) between the original and a new transform, the same postsynaptic neuron(s) will be excited and so increase their synaptic strengths to the neurons of the current transform. This process can continue across a series of overlapping transforms until they are all mapped onto the same output cells. Since similar images are more likely to be transforms of the same object than different stimuli, the CT mechanism provides an explanation for how transformation-invariant representations may develop in the ventral visual system.

In this set of simulations, the parameters were chosen to encourage the operation of the CT learning mechanism (Stringer et al., 2006) while excluding any trace-like effects (Földiák, 1991). To this end, spatial overlap between successive transforms was generally kept high (13 transforms per stimulus each covering 56 neurons and shifting by 12 neurons per transform by default). Also a short time constant of 2ms was used for the Excitatory-Excitatory (feed-forward) synaptic conductances, τ_*EE*_. These conditions were hypothesised to support a CT-like learning mechanism in a spiking neural network.

#### 3.1.1. Invariance learning with CT

**Figure 3:**
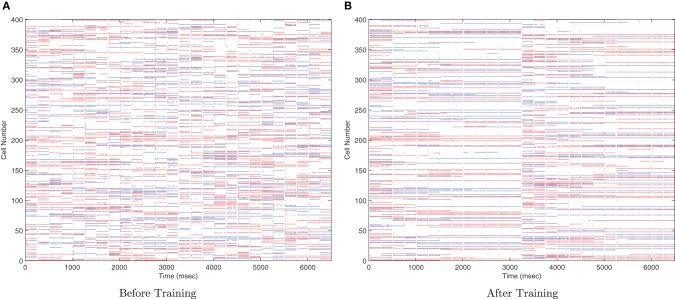
**Raster plots of output layer cells before and after training from the CT baseline simulation.** Before training **(A)** the output cells respond randomly to transforms of each stimulus. After training **(B)** the raster plot shows cells sensitive to all transforms of stimulus one (0–3250ms) and other cells sensitive to all transforms of stimulus two (3250–6500ms).

**Figure 4:**
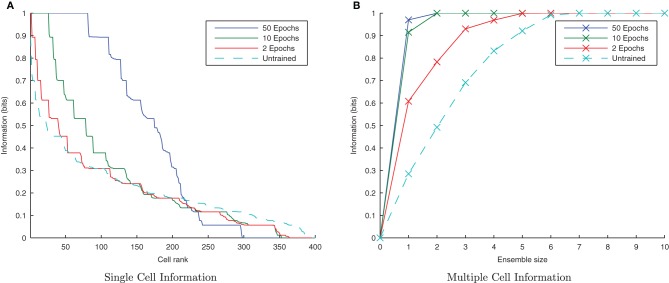
**Baseline demonstration of translation-invariant representations for four degrees of training: untrained, 2 epochs, 10 epochs, and 50 epochs.** The single cell information analysis **(A)**, shows that for 10 or more epochs of training, approximately 40 output cells have achieved a very high information content and the multiple cell information plot **(B)** confirms that both of the stimuli are represented by cells which are exclusively tuned to one stimulus or the other.

This simulation demonstrates the formation of transformation-invariant representations in the output cells through STDP as illustrated by the raster plots in Figure 3 (which contrast the untrained with the trained network) and the information plots of Figures 4A and B. The level of inhibition had to be tuned so that the spikes from additional neurons from successive transforms (in the input layer) could be brought into phase with those already firing from the previous transforms. While the feed-forward excitatory weights were plastic and hence modified through learning, their maximum level was set to 4 nS to achieve a reasonable level of output layer activity for the network size and connectivity. It can be seen from the pre-training raster plot (Figure 3A) that before learning, output neurons respond to a random set of transforms of each stimulus. However, post-training (Figure 3B), there are several cells which are responsive across the whole set of transforms for the first stimulus which are presented contiguously over the first 3250ms, and several other cells which respond to all transforms of the second stimulus presented contiguously over the second 3250ms.

In accordance with the raster plot, the *I*(*s*,*R*) (single-cell information measure) plots show many more cells in the network have attained the maximum information content (1 bit) than in the untrained case, demonstrating both transformation-invariance and stimulus specificity. Examining the *I*(*s*,*s*′) (multiple cell information measure) plots shows that the maximum information about the stimulus set *S* is reached with fewer than the 10 available cells of the output ensemble of the highest scoring cells (in terms of their *I*(*s*,*R*) values), thus confirming that both stimuli are represented invariantly.

#### 3.1.2. Temporal specificity

**Figure 5:**
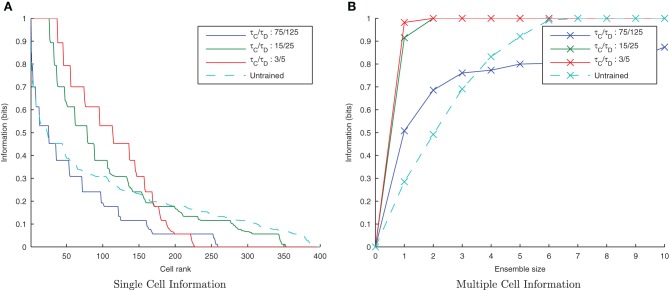
**Information plots of varying STDP time constants, τ_*C*_ and τ_*D*_.** With shorter time constants the single cell information content **(A)** is seen to increase as learning becomes more temporally specific. The multiple cell information **(B)** demonstrates that for short plasticity time constants, both stimuli are represented by the ensemble of output cells. STDP (with sufficiently temporally specific time constants) together with the synchronization of neuronal firing is here able to facilitate the learning of transformation-invariant representations.

By default, the learning time constants, τ_*C*_ and τ_*D*_, used in these simulations are 15 and 25ms in accordance with Perrinet (2003). Here we reran the same simulations but shortened or lengthened these time constants by a factor of five (maintaining the same 3/5 ratio) to give 3/5ms and 75/125 ms for τ_*C*_/τ_D_, respectively. Figure 5A shows a trend of a much greater information content in the network with the shorter (more temporally specific) time constants (3/5ms) with the accompanying *I*(*s*,*s*′) plot confirming that both stimuli are being represented (see Figure 5B). Network performance drops, however, with the longer (less temporally specific) STDP time constants 75/125 as the learning rule is less capable of capturing the temporally specific causal relationship of the input/output spike volleys.

**Figure 6:**
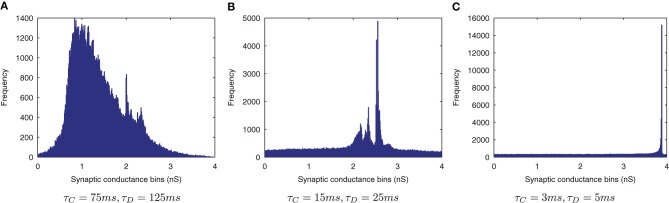
**Synaptic Weight Distributions of varying STDP time constants, τ_*C*_ and τ_*D*_.** Compared to the standard case τ_*C*_ = 15ms, τ_D_ = 25ms **(B)** longer plasticity time constants τ_*C*_ = 75ms, τ_D_ = 125ms **(A)** result in a smoother, more distributed profile of synaptic weights. With shorter time constants τ_*C*_ = 3ms, τ_D_ = 5ms **(C)** the distribution is seen to become more peaked with larger synaptic weights as learning becomes more temporally specific and the weight updates experience more LTP.

The effect of shortening the STDP time constants is that after a pre-post spike pairing results in LTP, the following presynaptic spike from the next wave comes a relatively long time after the initial pair, such that the effect of its post-pre LTD is significantly lessened. The synaptic weight distributions in Figure 6 support this, exhibiting a peaked distribution of synaptic efficacies arising from the initially flat uniform distribution (as expected from a multiplicative model of STDP in the standard case, τ_*C*_=15 ms, τ_*D*_=25 ms, Figure 6B) and more peaked distributions with shorter STDP time constants (Figure 6C) indicating more specific learning. The higher proportions of large weights with the shorter learning time constants are what might be expected from an unbalanced learning rule dominated by LTP when waves of input spikes are widely spaced relative to the time delay until the postsynaptic spikes which they cause. In contrast, the weight distribution with the longer STDP time constants is smoother, indicating a less trained layer of synaptic weights (Figure 6A).

#### 3.1.3. Lateral inhibition and synchrony

From earlier simulations, it is apparent that this training paradigm and the STDP model are very sensitive to the effects of the strength of inhibition on the synchronization of input spikes. We therefore systematically varied the strength of Δ*g*_*IE*_, the *Inhibitory* → *Excitatory* conductances (which were non-plastic) to understand these effects in more detail.

**Figure 7:**
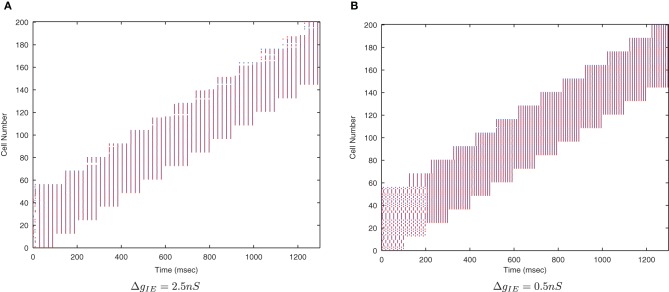
**Raster plots of the inputs (for all transforms of Stimulus 1 only) presented during training for two levels of inhibitory conductance.** The raster plot produced with the standard inhibitory conductance strength of Δ*g*_*IE*_ = 2.5 nS shows synchronised input volleys across all transforms of the stimulus **(A)**. It can be seen at the lower inhibitory strength (Δ*g*_*IE*_ = 0.5 nS) that the neurons within some of the transforms become desynchronized with respect to one another **(B)**.

**Figure 8:**
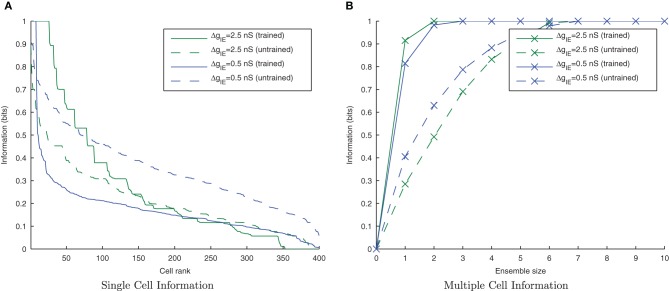
**Single and Multiple cell information plots for different degrees of inhibitory strength.** As the inhibitory strength decreases, the information in the network declines, as shown in the single cell **(A)** and multiple cell **(B)** information measures. This is due to the increased difficulty for the whole set of neurons representing a particular transform of a stimulus to fire in synchrony.

Figure 7 shows that as the level of inhibition is reduced and the cell membrane potential noise begins to cause jitter in the spike timings, the new input layer neurons from successive transforms no longer fire in phase with those neurons from previous transforms. This reduces invariance learning in the output layer, where the information content can also be seen to be reduced (Figure 8).

#### 3.1.4. Degree of overlap

From previous rate-coded simulations it is clear that CT learning requires a high degree of resemblance among adjacent members of a set of transforms in order to associate them together. If this mechanism is being employed in the present spiking model, its performance should suffer by reducing this transform similarity. This was tested by removing intermediate transforms leaving only every *2nd* or *3rd* transform from the original sets of 13 transforms per stimulus (with a consecutive transform overlap of 44 neurons) such that there were only 7 or 5 transforms per stimulus, respectively. Since they still occupied the same proportion of the input layer, the degree of overlap between any two consecutive transforms was correspondingly lower, being 32 or 20 neurons, respectively.

**Figure 9:**
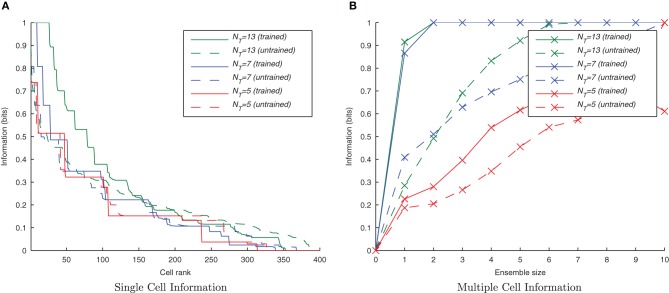
**Information plots for three different degrees of overlap between successive transforms.** The information content of the output cells can be seen to fall, in the single cell measure **(A)** and the multiple cell measure **(B)** as the degree of overlap is reduced. This is a property of CT learning which requires a sufficient degree of spatial overlap between transforms to build invariance.

It can be seen from Figure 9 that by reducing the spatial overlap between successive transforms of each object, the information content of the output layer declines (despite there being fewer transforms to associate together) since there are fewer cells that respond invariantly across all transforms of a given stimulus. This confirms that the network is learning invariance by a spiking equivalent of the CT learning mechanism.

#### 3.1.5. Interleaved transforms

Since the degree of similarity between any two transforms of a stimulus is the same regardless of when they are presented to the network, under a CT learning regime it should not matter whether the transforms are seen close together in time or not. One of the key properties of CT learning is therefore its ability to enable a network to learn about stimuli, even when their transforms are interleaved with those of another stimulus (analogous to learning to recognize two faces or objects as the viewer saccades back and forth between them). To test this hypothesis we presented transforms of each stimulus alternately i.e., *S*^*t*_1_^_1_, *S*^*t*_1_^_2_, *S*^*t*_2_^_1_, *S*^*t*_2_^_2_, …, *S*^*t*_*n*_^_1_, *S*^*t*_*n*_^_2_. If neurons are able to develop transformation-invariant responses with this training paradigm, it proves the learning mechanism is not utilizing a temporal trace.

**Figure 10:**
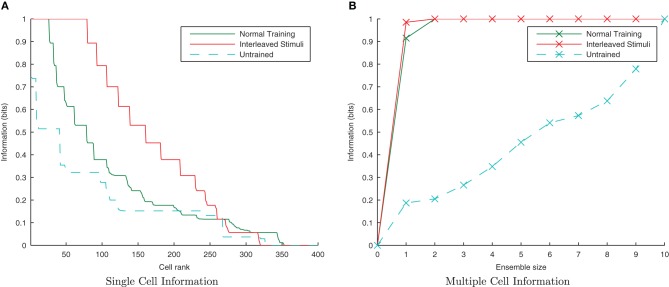
**Information plots showing the difference in training the network normally and by interleaving transforms of both stimuli.** By interleaving the collections of transforms, the single cell information content has not declined **(A)** while the multiple cell information **(B)** confirms that both stimuli are still represented. This is a property of CT learning, which does not require different transforms of a stimulus to be seen consecutively in time.

Here it is evident from the information analysis (Figure 10) that the network has managed to learn about the individual stimuli with this additional constraint. Both the single and multiple information measures show not just comparable results to the consecutive presentation of each stimulus's transforms during training but surprisingly, an improvement over the standard case. Examining the input layer rasters, this enhancement to learning from interleaving the stimuli seems to be due to the fact that under normal training, the first one or two spike volleys of a new transform have not yet recruited the additional neurons (which were not part of the previous transform) due to the lateral inhibition suppressing them. In contrast, neurons in the overlapping region are under constant stimulation from the injected current and so continue to fire, whereas those neurons exclusive to the previous transform stop firing when no longer stimulated with direct current.

When the stimuli are interleaved, however, all of the input neurons representing the new transform are stimulated by current injection at the same time (rather than their cell membrane potentials starting at different points in the stimulation cycle) and so fire simultaneously from the very first volley. Using a stimulating direct current of 1 nA with the cell body parameters and network connectivity given in Table 1, the neurons will fire approximately five complete volleys of spikes in the 100ms presentation period (50Hz). The ultimate effect of this training difference is that in the interleaved case, each transform will be represented by five complete spike volleys (as opposed to only three of four in the standard case) and hence will be trained more fully (with more useful weight updates) over the same training duration.

#### 3.1.6. Randomized transform order

CT learning is also able to form transformation-invariant representations when the individual transforms of an object are presented in a random order during training. This is analogous to learning to recognize a face or object from a number of random “snapshot” views rather than seeing it move smoothly. The consequence of such a training regime is that there is not necessarily any overlap between two consecutive transforms in time. At the beginning of training when the feed-forward weights are randomly initialized, this training regime may mean that different output neurons learn to respond to different subsets of each stimulus' transforms, thus making it harder for the similarity-based CT mechanism to associate all related (overlapping) transforms together onto the same output neurons. If, however, there is a sufficient number of such training epochs and degree of competition in the output layer, eventually each transform will be randomly followed by a similar enough transform such that the same postsynaptic cell is fired which eventually learns invariance across the whole set of transforms.

**Figure 11:**
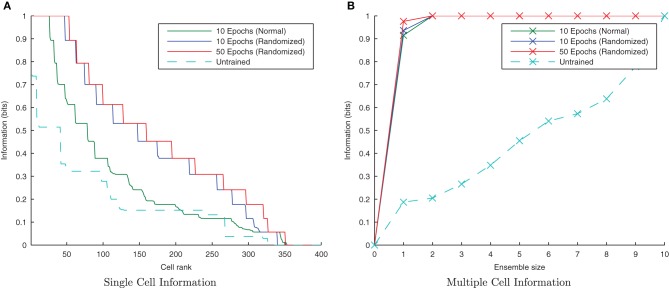
**Information plots showing the effect of training the network by randomizing the order of transforms.** Despite this paradigm representing more difficult learning conditions for a CT mechanism, the single cell **(A)** and multiple cell information measures **(B)** demonstrate good performance. CT learning is thus able to build invariance if enough overlapping transforms are presented to the network over time, irrespective of the temporal sequence they are presented in.

Initially randomizing the order of transforms degraded the network performance as expected. However, building upon the learning enhancement found in the previous simulations with interleaving the stimuli, simulations were repeated with simultaneously randomized transform order and interleaved stimuli. Figure 11 demonstrates that the network is able to cope with randomizing the order of the transforms.

### 3.2. Trace learning

Trace learning utilizes the temporal continuity of objects in the world to learn transformation-invariant representations (Földiák, 1991). The mechanism relies upon the proposal that over short time scales, successive images are more likely to be transforms of the same object rather than different objects. The trace learning rule (Földiák, 1991; Wallis and Rolls, 1997) uses these temporal statistics of visual input by incorporating a temporal trace of the previous (typically postsynaptic) neural activity into a simple Hebbian learning rule, which helps to maintain firing in the same output cell(s) when successive transforms are presented. Through further Hebbian synaptic modifications, successive transforms may become associated together onto the same output cells leading to transformation-invariant neurons.

In contrast to the CT simulations (section 3.1), here we lengthen the synaptic time constant τ_*EE*_ to 150ms to explore the hypothesis that by continuing to bleed current into a postsynaptic neuron, the activity generated by one transform may be associated with the next. In this way, a temporal trace effect may be achieved, allowing a spiking neural network to learn through temporal rather than spatial continuity.

#### 3.2.1. Invariance learning with a temporal trace

This simulation demonstrates the formation of transformation-invariant representations in the output cells through STDP and a trace-like effect from longer *E*→ *E* synaptic time constants. The other parameters remained the same as in the CT simulations except that the maximum strength of the plastic feed-forward excitatory synapses was reduced to 1.25 nS (from 4 nS) to compensate for the greater degree of excitation arising from the longer feed-forward synaptic time constant. Also the stimuli were changed such that in the following trace simulations, there are 10 transforms per stimulus (consisting of 20 neurons each) which are shifted by 20 neurons for each transform such that there is no spatial overlap between transforms. Since these transforms are orthogonal, any CT effects from spatial overlap are eliminated. Additionally, since the spatio-temporal statistics of natural stimuli tend to have different transforms of the same stimulus closer together in time more frequently than transforms of different stimuli, the neurons were allowed to settle between presentation of the two sets of transforms (stimuli) to effectively reduce the temporal continuity between different stimuli so as to avoid introducing an artificial trace effect between them. These changes allow for a controlled investigation of whether orthogonal transforms may be linked together by a trace-like learning mechanism by lengthening the excitatory synaptic conductance.

**Figure 12:**
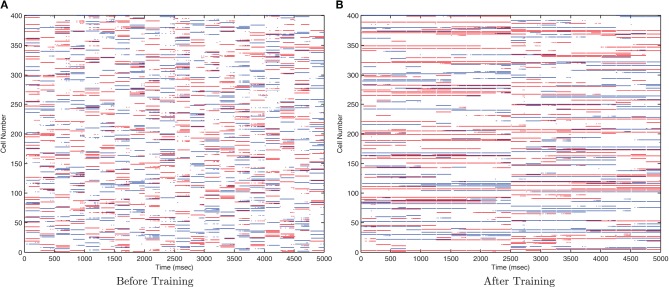
**Raster plots of output layer cells before and after training from the trace baseline simulation.** Before training **(A)** the output cells respond to random subsets of transforms of each stimulus. After training **(B)** some cells are sensitive to most or all transforms of Stimulus 1 (0–2500ms), while other output cells are sensitive to most or all transforms of Stimulus 2 (2500–5000ms).

**Figure 13:**
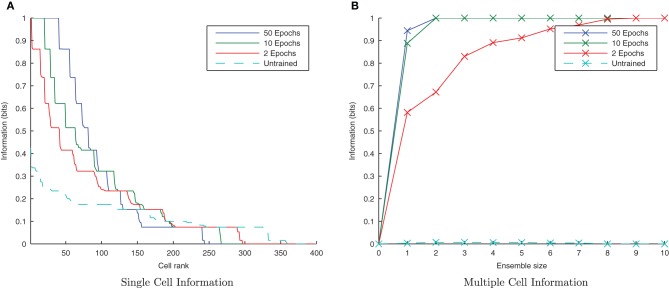
**Baseline demonstration of translation-invariant representations for varying levels of training.** The single cell information analysis **(A)** shows fewer maximally informative cells than in the CT simulations but the multiple cell information plot **(B)** confirms that both stimuli are represented by cells which are exclusively tuned to one stimulus or the other.

Due to the random initialization of the feed-forward weights, output neurons before training respond to a random set of transforms of each stimulus (Figure 12A), whereas after training Figure 12B shows both stimuli are represented by cells which are invariant to most transforms of their respective stimuli, while the information plots (Figure 13) confirm that both stimuli may be identified with a small ensemble of output neurons. In earlier simulations without allowing the neurons to settle between each set of transforms (not shown here), the multiple cell information measure was found to drop with further training. This was caused by the association of the two stimuli together since they are presented consecutively in time during training with long synaptic time constants, so the last transform of the first stimulus was still active as the first transform of the second stimulus was presented, thereby leading to their association.

#### 3.2.2. Temporal specificity

Lengthening the synaptic conductance time constant, τ_*EE*_, may affect the dynamics of synaptic plasticity in unforseen ways, so it was important to explore a range of values for the plasticity time constants as for the first set of CT simulations. As before, τ_*C*_=15 ms and τ_*D*_=25ms were used as standard for the learning time constants but here they are shortened and lengthened by a factor of five (keeping the same 3/5 ratio) for comparison (while τ_*EE*_ remains fixed at 150ms).

**Figure 14:**
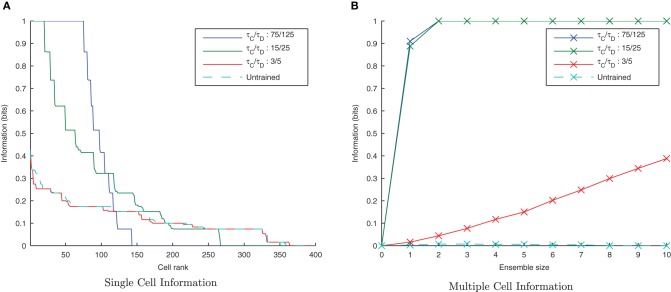
**Information plots of varying STDP time constants, τ_*C*_ and τ_*D*_.** Both single cell **(A)** and multiple cell **(B)** information measures show an increase in network performance with longer, less temporally specific STDP time constants and a decrease in performance with shorter STDP time constants. This is the reverse of the trend found in the equivalent CT simulations.

**Figure 15:**
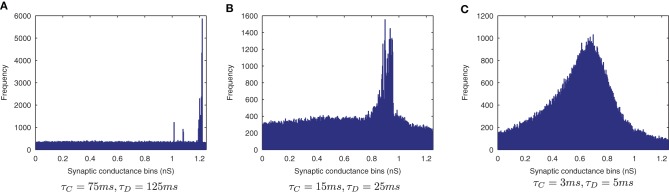
**Synaptic Weight Distributions of varying STDP time constants.** In contrast to the standard case {τ_*C*_ = 15ms, τ_*D*_ = 25ms} **(B)** the more peaked distribution for longer STDP time constants {τ_*C*_ = 75ms, τ_*D*_ = 125ms} indicates more useful learning under this regime **(A)**. Contrary to the equivalent CT simulations, the use of shorter STDP time constants {τ_*C*_ = 3ms, τ_*D*_ = 5ms} **(C)** yields a smoother, less trained profile.

The results are shown in the information plots of Figure 14 and the synaptic weight distributions of Figure 15. In contrast to the previous CT simulations, the network performance degrades with more temporally specific (shorter) STDP time constants (Figure 14) but improves with longer, less specific STDP time constants (the reverse trend). Similarly, this opposite trend is borne out by the synaptic weight distributions (Figure 15) exhibiting a smoother profile (indicating less useful training) for short STDP time constants and a more peaked profile for longer, less temporally specific STDP time constants.

This reverse effect may be understood in the context of the two learning mechanisms whereby CT learning performs best with tightly synchronized, temporally-specific causal spike volleys, hence a temporally specific form of STDP is most appropriate. In contrast, trace learning requires activity to continue over an extended period of time between different transforms in order to associate them together, and as such the relationship it needs to capture is less temporally specific and thus a less specific form of STDP is better suited for this purpose.

#### 3.2.3. Lateral inhibition and synchrony

**Figure 16:**
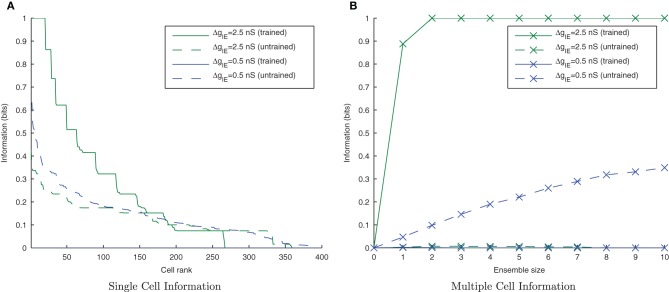
**Single and Multiple cell information plots for different degrees of inhibitory strength.** As the inhibitory strength decreases the information in the network declines, exemplified in both single **(A)** and multiple cell **(B)** information measures.

As the level of inhibition is reduced, and the effects of timing jitter from the cellular membrane potential noise become more prominent, the new input layer neurons from successive transforms no longer fire in phase with those neurons from previous transforms and the information content of the output layer (Figure 16) can be seen to be reduced.

#### 3.2.4. Interleaved transforms

By interleaving transforms of the two stimuli alternately through time, transforms from different stimuli should be associated together by their temporal continuity with a trace-like mechanism. Unlike in the previous CT simulations (where the association is not time-dependent, only similarity dependent), this inter-stimulus association should lead to a large drop in information since the network will be unable to distinguish between the two stimuli. The neurons were not allowed to settle between presentations of different stimuli (as with previous trace simulations) as this would negate the effect of interleaving the stimuli and undermine the purpose of this section of simulations.

**Figure 17:**
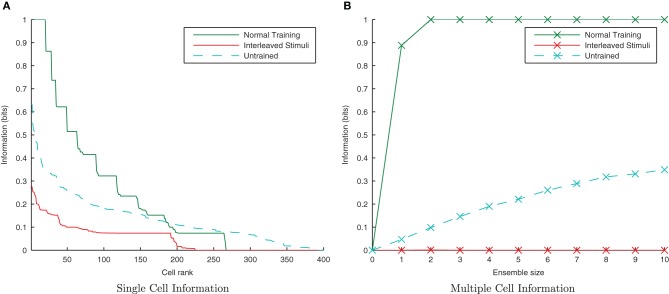
**Information plots showing the difference in training the network normally and by interleaving transforms of both stimuli.** By interleaving the collections of transforms, the single cell **(A)** and multiple cell **(B)** information measures indicate that performance has dropped to lower levels than obtained with a random (untrained) network. This is a typical property of Trace learning and is in marked contrast to the equivalent CT learning results with short synaptic time constants.

From Figure 17 it is evident that interleaving the transforms of the two stimuli has significantly reduced the information content of the network as expected. In the interleaved case, the single-cell information content (Figure 17A) has dropped to a poorer level than the untrained case (tested with a random uniform distribution of synaptic weights) as transforms from each stimuli have been associated together, meaning the output cells are less able to discriminate between stimuli than in their initial untrained, random state. From the multiple-cell information plot (Figure 17B) it is clear that virtually all transforms of all stimuli have become associated together since even using the ten best single-cell information neurons barely raises the multiple cell information measure above 0-bits since the cells are unable to discriminate one stimulus from the other.

#### 3.2.5. Randomized transform order

If the network is using a temporal trace to associate orthogonal transforms together, randomizing the order of those transforms within a stimulus block, but still presenting all transforms of one stimulus followed by all transforms of the other, should not significantly degrade its performance. Moreover, there is good reason to expect that the performance should be improved slightly, as this training paradigm will help to associate each transform, *S*^*t*_*n*_^_1_, with each other from the same stimulus rather than just its neighboring transforms, *S*^*t*_*n*−1_^_1_ and *S*^*t*_*n*+1_^_1_.

**Figure 18:**
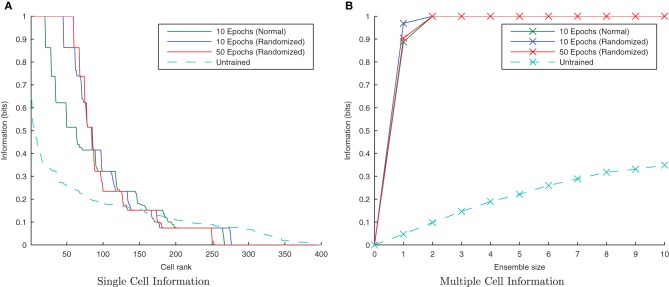
**Information plots showing the difference in training the network by presenting the transforms sequentially and by randomizing the order of transforms.** It is evident from the single cell **(A)** and multiple cell **(B)** information measures that trace learning exhibits better performance with randomized transformation order. This is because under the randomised training regime, there is greater scope for associations to be made between more pairs of transforms (rather than each with just its sequential neighbors) over the course of many training epochs.

It is clear from Figure 18 that with the longer synaptic conductance time course (τ_*EE*_=150ms) and the same degree of training, the randomized transform case has performed better than the standard non-randomized paradigm as expected, improving further with more epochs of training. In previous simulations this training paradigm proved difficult for the CT mechanism with an initially random set of feed-forward weights, since different pools of output neurons were stimulated by randomly ordered transforms (due to having less spatial overlap between consecutive transforms on average). In the case of randomly ordered transforms with the trace learning mechanism, however, the lower degree of spatial overlap is irrelevant as the same pool of output neurons is kept active for all the transforms of a particular stimulus by virtue of the longer synaptic time constants and the consecutive presentation of all transforms of a particular stimulus (albeit not necessarily in order).

## 4. Discussion

In the above simulations we have shown that a biologically realistic spiking neural network with STDP can operate in two very different ways to achieve transformation-invariant representations. These simulations lend more biological plausibility to the Trace and CT learning mechanisms, which may be utilized by the same model with slight differences in the training environment or the physical parameters of the neurons.

With short synaptic conductance time constants between the pyramidal neurons (τ_*EE*_), the model works similarly to the CT learning mechanism. In this case, the network requires the transforms to be spatially overlapping (as a direct consequence of the learning mechanism) but can cope with interleaving the transforms of different stimuli and thus bears the characteristics of the equivalent rate-coded mechanism (Stringer et al., 2006). Importantly, this mechanism is sensitive to the strength of lateral inhibition, which under optimal conditions serves to maintain the synchronous firing of neurons representing the novel part of an unseen transform with those already potentiated from previous learning of another transform. Without this effect of lateral inhibition, these novel neurons will most likely fire outside the time window for significant LTP, and may possibly come after the postsynaptic neuron has fired leading to LTD.

Lengthening the very same synaptic conductance time constants (τ_*EE*_), enables the model to work with a Trace learning mechanism. In this case the network uses temporal continuity to associate together orthogonal (completely non-overlapping) transforms and consequently fails to develop invariance and stimulus specificity if the transforms of different stimuli are interleaved. While these properties are the same as for the classic McCulloch-Pitts neuron, it is interesting to note that in such a rate-coded model, the trace term is associated with the presynaptic or (more commonly) postsynaptic *neuron* (Rolls and Milward, 2000). In contrast, in a conductance-based spiking neural network, the trace can instead be associated with the individual *synapses* between two connected neurons. This is a measurable property of biological neurons and suggests where to focus neurophysiological investigation aiming to understand invariance learning mechanisms.

While the Trace and CT learning mechanisms have been studied here in isolation, it seems likely that a combination of both would be employed to varying degrees depending upon the statistics of the inputs to each layer of the brain. In early layers (e.g., V1), the patterns of stimulation are likely to change more from one transform to another since the neurons here are highly specific in their sensitivity to a location and orientation. In later layers, however, such as Inferotemporal cortex (IT), the invariance built in the earlier layers will mean that inputs to these cells are less changeable from one transform to another. Having passed through several layers of pyramidal cells with lateral inhibition acting at each stage, the spike volleys representing a stimulus may also become more synchronized (Diesmann et al., 1999). Under these conditions, we therefore expect that as the similarity between transforms increases through the layers, the CT mechanism will become more prominent and trace effects will become less important, which would be evidenced by progressively quicker synaptic conductance decays (shorter time constants).

If it is the case that the ventral visual system uses an effective synaptic time constant between the two extremes presented in the simulations here, we would therefore predict that the type of learning occurring for any given stimuli would be highly dependent on how those stimuli are presented, for example with rapidly transforming (and hence spatially dissimilar successive views) leading to more of a Trace learning regime, whereas temporally separate exposures would require a high degree of similarity between the views for the CT mechanism to work.

The work presented here is a first step toward understanding how the Trace and CT learning rules may be utilised in a spiking neural network, and as such will naturally have limitations. So far, the model has been presented with orthogonal, non-overlapping “toy” stimuli rather than the more distributed, spatially overlapping stimuli found in the natural world. Whilst we acknowledge that these highly idealized representations are somewhat lacking in ecological validity, they were employed in order to isolate each learning mechanism in a precise and identifiable way. Further work would benefit however from exploring these learning mechanisms with more natural, spatially overlapping stimuli.

A further limitation concerns the Trace learning mechanism. By lengthening the time constant of the feed-forward synaptic conductances, τ_*EE*_, the excitatory activity reaching the output neurons decays more slowly and results in much higher firing rates in the output neurons (approximately 200 spikes/s) than in the CT simulations (approximately 50 spikes/s). While these rates are still within the realms of biological plausibility, they are towards the edge of it and so the conclusions would be on firmer ground through exploring additional mechanisms to reduce these high firing rates.

### 4.1. Future directions

In understanding the dynamics of learning transformation-invariant representations in spiking neural networks, we have only demonstrated *translation* invariance so far. A natural extension to this body of work would therefore be to investigate this learning process with with other kinds of transforms commonly found in natural visual scenes and investigated in rate-coded models including, for example, rotations (Stringer et al., 2006), occlusions (Stringer and Rolls, 2000) and changes in scale (Wallis and Rolls, 1997). This would provide a more general understanding of the variations in the problems of visual object recognition that the visual system must overcome.

Furthermore, the use of realistic 3D shapes and faces will also allow the model to be more directly compared to psychophysical data, both in terms of the effects on representations formed from exposure to realistic images (Simoncelli, 2003; David et al., 2004; Felsen and Dan, 2005; Felsen et al., 2005) and testing if invariance learning may be achieved at natural speeds of transformation (e.g., rotation). Neuronal parameters such as the synaptic time constants (e.g., τ_*EE*_) and the learning time constants (τ_*C*_ and τ_*D*_) may be crucial to invariance learning with realistic stimuli. Exploring the interaction between the speed of transformation of objects and the parameters of the model should lead to concrete predictions which may be tested against neurophysiological data. For example this may reveal an upper-threshold of stimulus movement speed which still allows transformation-invariant representations to form, or even that our visual systems typically use a number of static views to learn invariance.

Natural stimuli will also test the model's ability to learn transformation-invariant representations with effectively distributed, overlapping representations rather than the orthogonal non-overlapping representations employed so far. This would mean that the network could no longer appear to solve the problem through learning about retinal location.

In addition to enhancing the ecological validity of the stimuli and their presentation paradigm, the model itself could be modified to incorporate additional features found in its biological counterpart including lateral excitatory connectivity, cell firing-rate adaptation and multiple layers of feed-forward weights, some or all of which may prove to be necessary for solving the more complex invariance learning problems, for instance, with natural scenes composed of multiple objects.

## 5. Conclusion

In the work presented here, we have demonstrated how a spiking neural network may exhibit two very different modes of invariance learning, which share the characteristic properties of their rate-coded counterparts. This was achieved in a single model by changing, (most notably), the time constant of the feed-forward synaptic conductances and the properties of the stimulus sets. Through developing more biologically accurate spiking models in this way, we may build upon incites from previous work to more fully understand the detailed mechanisms of visual invariance learning in the brain.

### Conflict of interest statement

The authors declare that the research was conducted in the absence of any commercial or financial relationships that could be construed as a potential conflict of interest.

## Footnotes

^1^These early neuron models were designed to show that the elementary components of the brain could compute elementary logic functions. The belief commonly held at the time being that intelligence is based upon symbolic reasoning, which in turn rests upon the foundations of logic.

^2^Typically, only 100–150ms is required to respond to complex stimuli.

^3^For a general introduction to Information Theory see MacKay, 2003.

## References

[B1] Abbott L. F. Nelson S. B. (2000). Synaptic plasticity: taming the beast. Nat. Neurosci. 3, 1178–1183 10.1038/81453 11127835

[B2] Amit D. J. Brunel N. (1997). Dynamics of a recurrent network of spiking neurons before and following learning. Network 8, 373–404

[B3] Bell A. J. Sejnowski T. J. (1997). The “independent components” of natural scenes are edge filters. Vision Res. 37, 3327–3338 10.1016/S0042-6989(97)00121-1 9425547 PMC2882863

[B4] Bi G-Q. Poo M-m. (1998). Synaptic modifications in cultured hippocampal neurons: dependence on spike timing, synaptic strength, and postsynaptic cell type. J. Neurosci. 18, 10464–10472 9852584 10.1523/JNEUROSCI.18-24-10464.1998 PMC6793365

[B5] Bialek W. Rieke F. de Ruyter van Steveninck R. R. Warland D. (1991). Reading a neural code. Science 252, 1854–1857 2063199 10.1126/science.2063199

[B6] Booth M. C. A. Rolls E. T. (1998). View-invariant representations of familiar objects by neurons in the inferior temporal visual cortex. Cereb. Cortex 8, 510–523 10.1093/cercor/8.6.510 9758214

[B7] Dan Y. Poo M-m. (2006). Spike timing-dependent plasticity: from synapse to perception. Physiol. Rev. 86, 1033–1048 10.1152/physrev.00030.2005 16816145

[B8] David S. V. Vinje W. E. Gallant J. L. (2004). Natural stimulus statistics alter the receptive field structure of v1 neurons. J. Neurosci. 24, 6991–7006 10.1523/JNEUROSCI.1422-04.2004 15295035 PMC6729594

[B9] Desimone R. (1991). Face-selective cells in the temporal cortex of monkeys. J. Cogn. Neurosci. 3, 1–8 10.1162/jocn.1991.3.1.1 23964801

[B10] Diesmann M. Gewaltig M. O. Aertsen A. (1999). Stable propagation of synchronous spiking in cortical neural networks. Nature 402, 529–533 10591212 10.1038/990101

[B11] Elliffe M. C. M. Rolls E. T. Stringer S. M. (2002). Invariant recognition of feature combinations in the visual system. Biol. Cybern. 86, 59–71 11924570 10.1007/s004220100284

[B12] Felsen G. Dan Y. (2005). A natural approach to studying vision. Nat. Neurosci. 8, 1643–1646 10.1038/nn1608 16306891

[B13] Felsen G. Touryan J. Han F. Dan Y. (2005). Cortical sensitivity to visual features in natural scenes. PLoS Biol. 3:e342. 10.1371/journal.pbio.0030342 16171408 PMC1233414

[B14] Ferster D. Spruston N. (1995). Cracking the neuronal code. Science 270, 756–757 10.1126/science.270.5237.756 7481761

[B15] Földiák P. (1991). Learning invariance from transformation sequences. Neural Comput. 3, 194–200 10.1162/neco.1991.3.2.194 31167302

[B16] Földiák P. (1993). The ‘ideal homunculus’: statistical inference from neuronal population responses, in Computation and Neural Systems, eds Eeckman F. H. Bower J. M. (Norwell, MA: Kluwer Academic Publishers), 55–60

[B17] Fries P. Schröder J. H. Roelfsema P. R. Singer W. Engel A. K. (2002). Oscillatory neuronal synchronization in primary visual cortex as a correlate of stimulus selection. J. Neurosci. 22, 3739–3754 11978850 10.1523/JNEUROSCI.22-09-03739.2002 PMC6758402

[B18] Fukushima K. (1988). Neocognitron: a hierarchical neural network capable of visual pattern recognition. Neural Netw. 1, 119–130

[B19] Gerstner W. Kistler W. (2006). Spiking Neuron Models: Single Neurons, Populations, Plasticity, 3rd Edn Cambridge, UK: Cambridge University Press

[B20] Hebb D. O. (1949). The Organization of Behaviour: A Neuropsychological Theory. New York, NY: Wiley

[B21] Hubel D. H. Wiesel T. N. (1968). Receptive fields and functional architecture of monkey striate cortex. J. Physiol. 195, 215–243 4966457 10.1113/jphysiol.1968.sp008455 PMC1557912

[B22] Ito M. Tamura H. Fujita I. Tanaka K. (1995). Size and position invariance of neuronal response in monkey inferotemporal cortex. J. Neurophysiol. 73, 218–226 7714567 10.1152/jn.1995.73.1.218

[B23] Izhikevich E. M. (2003). Simple model of spiking neurons. IEEE Trans. Neural Netw. 14, 1569–1572 Available online at: http://vesicle.nsi.edu/users/izhikevich/publications/whichmod.htm 10.1109/TNN.2003.820440 18244602

[B24] Kreiter A. K. Singer W. (1996). Stimulus-dependent synchronization of neuronal responses in the visual cortex of the awake macaque monkey. J. Neurosci. 16, 2381–2396 8601818 10.1523/JNEUROSCI.16-07-02381.1996 PMC6578521

[B25] Kuwabara N. Suga N. (1993). Delay lines and amplitude selectivity are created in subthalamic auditory nuclei: the brachium of the inferior colliculus of the mustached bat. J. Neurophysiol. 69, 1713–1724 8389837 10.1152/jn.1993.69.5.1713

[B26] Maass W. Bishop C. M. (eds.) (1999). Pulsed Neural Networks. Cambridge, MA: MIT Press

[B27] MacKay D. J. C. (2003). Information Theory, Inference and Learning Algorithms. Cambridge, CA: Cambridge University Press

[B28] Markram H. Lübke J. Frotscher M. Sakmann B. (1997). Regulation of synaptic efficacy by coincidence of postsynaptic APs and EPSPs. Science 275, 213–215 10.1126/science.275.5297.213 8985014

[B29] Masquelier T. Hugues E. Deco G. Thorpe S. J. (2009). Oscillations, phase-of-firing coding, and spike timing-dependent plasticity: an efficient learning scheme. J. Neurosci. 29, 13484–13493 10.1523/JNEUROSCI.2207-09.2009 19864561 PMC6665016

[B30] McCormick D. A. Connors B. W. Lighthall J. W. Prince D. A. (1985). Comparative electrophysiology of pyramidal and sparsely spiny stellate neurons of the neocortex. J. Neurophysiol. 54, 782–806 2999347 10.1152/jn.1985.54.4.782

[B31] McCulloch W. Pitts W. (1943). A logical calculus of the ideas immanent in nervous activity. Bull. Math. Biol. 5, 115–133 2185863

[B32] Mel B. (1997). Seemore: combining color, shape, and texture histogramming in a neurally inspired approach to visual object recognition. Neural Comput. 9, 777–804 9161022 10.1162/neco.1997.9.4.777

[B33] Michler F. Eckhorn R. Wachtler T. (2009). Using spatiotemporal correlations to learn topographic maps for invariant object recognition. J. Neurophysiol. 102, 953–964 10.1152/jn.90651.2008 19494190

[B34] Op de Beeck H. Vogels R. (2000). Spatial sensitivity of macaque inferior temporal neurons. J. Comp. Neurol. 426, 505–518 10.1002/1096-9861(20001030)426:4<505::AID-CNE1>3.0.CO;2-M 11027395

[B35] Perrinet L. (2003). Comment Déchiffrer le Code Impulsionnel de la Vision? Étude du Flux Parallèle, Asynchrone et épars dans le Traitement Visuel Ultra-Rapide. Ph.D. thesis, Université Paul Sabatier, Toulouse, France

[B36] Perrinet L. Delorme A. Samuelides M. Thorpe S. J. (2001). Networks of integrate-and-fire neuron using rank order coding A: how to implement spike time dependent hebbian plasticity. Neurocomputing 38–40, 817–822

[B37] Quiroga R. Q. Reddy L. Kreiman G. Koch C. Fried I. (2005). Invariant visual representation by single neurons in the human brain. Nature 435, 1102–1107 10.1038/nature03687 15973409

[B38] Riesenhuber M. Poggio T. (1999). Hierarchical models of object recognition in cortex. Nat. Neurosci. 2, 1019–1025 10.1038/14819 10526343

[B39] Rolls E. T. Milward T. (2000). A model of invariant object recognition in the visual system: learning rules, activation functions, lateral inhibition, and information-based performance measures. Neural Comput. 12, 2547–2572 11110127 10.1162/089976600300014845

[B40] Rolls E. T. Treves A. (1998). Neural Networks and Brain Function. Oxford University Press, Oxford

[B41] Rolls E. T. Treves A. Tovee M. J. (1997). The representational capacity of the distributed encoding of information provided by populations of neurons in primate temporal visual cortex. Exp. Brain Res. 114, 149–162 10.1007/PL00005615 9125461

[B42] Rullen R. V. Thorpe S. J. (2001). Rate coding versus temporal order coding: what the retinal ganglion cells tell the visual cortex. Neural Comput. 13, 1255–1283 11387046 10.1162/08997660152002852

[B43] Šíma J. Orponen P. (2003). General-purpose computation with neural networks: a survey of complexity theoretic results. Neural Comput. 15, 2727–2778 10.1162/089976603322518731 14629867

[B44] Simoncelli E. P. (2003). Vision and the statistics of the visual environment. Curr. Opin. Neurobiol. 13, 144–149 10.1016/S0959-4388(03)00047-3 12744966

[B45] Stringer S. M. Perry G. Rolls E. T. Proske J. H. (2006). Learning invariant object recognition in the visual system with continuous transformations. Biol. Cybern. 94, 128–142 10.1007/s00422-005-0030-z 16369795

[B46] Stringer S. M. Rolls E. T. (2000). Position invariant recognition in the visual system with cluttered environments. Neural Netw. 13, 305–315 10.1016/S0893-6080(00)00017-4 10937964

[B47] Tanaka K. (1996). Representation of visual features of objects in the inferotemporal cortex. Neural Netw. 9, 1459–1475 10.1016/S0893-6080(96)00045-7 12662545

[B48] Tanaka K. Saito H. Fukada Y. Moriya M. (1991). Coding visual images of objects in the inferotemporal cortex of the macaque monkey. J. Neurophysiol. 66, 170–189 1919665 10.1152/jn.1991.66.1.170

[B49] Thorpe S. Fize D. Marlot C. (1996). Speed of processing in the human visual system. Nature 381, 520–522 10.1038/381520a0 8632824

[B50] Thorpe S. J. Delorme A. Van Rullen R. Paquier W. (2000). Reverse engineering of the visual system using networks of spiking neurons, in Proceedings. ISCAS 2000 Geneva. The 2000 IEEE International Symposium on Circuits and Systems (2000), Vol. 4, (Geneva), 405–408

[B51] Tovee M. J. Rolls E. T. Azzopardi P. (1994). Translation invariance in the responses to faces of single neurons in the temporal visual cortical areas of the alert macaque. J. Neurophysiol. 72, 1049–1060 7807195 10.1152/jn.1994.72.3.1049

[B52] Troyer T. W. Krukowski A. E. Priebe N. J. Miller K. D. (1998). Contrast-invariant orientation tuning in cat visual cortex: thalamocortical input tuning and correlation-based intracortical connectivity. J. Neurosci. 18, 5908–5927 9671678 10.1523/JNEUROSCI.18-15-05908.1998 PMC6793055

[B53] van Hateren J. H. van der Schaaf A. (1998). Independent component filters of natural images compared with simple cells in primary visual cortex. Proc. Biol. Sci. 265, 359–366 10.1098/rspb.1998.0303 9523437 PMC1688904

[B54] van Rossum M. C. Bi G-Q. Turrigiano G. G. (2000). Stable hebbian learning from spike timing-dependent plasticity. J. Neurosci. 8812–8821 11102489 10.1523/JNEUROSCI.20-23-08812.2000 PMC6773092

[B55] Wallis G. Rolls E. T. (1997). Invariant face and object recognition in the visual system. Prog. Neurobiol. 51, 167–194 10.1016/S0301-0082(96)00054-8 9247963

